# Intraindividual variability across cognitive tasks as a potential marker for prodromal Alzheimer’s disease

**DOI:** 10.3389/fnagi.2014.00147

**Published:** 2014-07-04

**Authors:** Andrea M. Kälin, Marlon Pflüger, Anton F. Gietl, Florian Riese, Lutz Jäncke, Roger M. Nitsch, Christoph Hock

**Affiliations:** ^1^Division of Psychiatry Research and Psychogeriatric Medicine, University of Zurich, ZurichSwitzerland; ^2^Department of Forensic Psychiatry, Psychiatric University Clinics, University of Basel, BaselSwitzerland; ^3^Division of Neuropsychology, Institute of Psychology, University of Zurich, ZurichSwitzerland; ^4^International Normal Aging and Plasticity Imaging Center, University of Zurich, ZurichSwitzerland; ^5^University Research Priority Program “Dynamics of healthy aging”, University of Zurich, ZurichSwitzerland

**Keywords:** Alzheimer’s disease, mild cognitive impairment, early diagnosis, cognitive control, cognitive variability, neuropsychology

## Abstract

Recent studies have shown that increased cognitive intraindividual variability (IIV) across accuracy scores from tests representing different cognitive domains (across-domain IIV) might indicate prodromal Alzheimer’s disease (AD). Although IIV has been proposed to index cognitive control processes, IIV across accuracy scores from cognitive control tasks (within-domain IIV) has not been examined in healthy controls subjects (HCS), mild cognitive impairment (MCI), and AD patients in a single comparative study. This study examines the discriminative properties of within-domain IIV, and across-domain IIV in 149 HCS, 31 MCI, and 26 AD. Three tasks representing different cognitive domains were identified to calculate across-domain IIV. Three other tasks representing cognitive control were identified to calculate within-domain IIV. The intraindividual standard deviation was calculated across accuracy scores. To compare IIV between groups, ANCOVAs with the covariates age, gender, education, and mean performance were computed. IIV scores in general were higher in AD vs. HCS (*p* < 0.01). Only across-domain IIV was higher in AD vs. MCI (*p* = 0.001), and only within-domain IIV was higher in MCI vs. HCS (*p* = 0.05). Within-domain IIV may constitute a cognitive marker for the detection of prodromal AD at the MCI stage, whereas across-domain IIV may detect beginning AD at the MCI stage.

## INTRODUCTION

The importance of reliable methods for the early detection of Alzheimer’s disease (AD) has increased with the expected availability of treatment methods, which may be most efficacious in preclinical (Masdeu et al., 2012) or early disease stages (Doraiswamy et al., 2002). Cognitive intraindividual variability (IIV) has evidenced representing a potential marker of early cognitive impairment (MacDonald et al., 2009), with IIV across multiple trials of a reaction time (RT) task or across different RT tasks (latency-based IIV) predicting global decline (Hultsch et al., 2002; Lövdén et al., 2007) and being increased in mild cognitive impairment (MCI; Duchek et al., 2009; McLaughlin et al., 2010), and AD (Hultsch et al., 2000; Jackson et al., 2012). However, integrating repetitive RT tasks into already existing comprehensive test batteries may increase the testing-associated burden on patients.

Considering IIV across accuracy scores of different cognitive tasks (accuracy-based IIV) may provide an alternative. Although latency- and accuracy-based IIV have reportedly been associated (Hultsch et al., 2002; Hilborn et al., 2009), the latter has not been studied extensively. However, the use of accuracy-based IIV across tests representing different cognitive domains (across-domain IIV) appears promising for predicting global (Kliegel and Sliwinski, 2004) and functional decline (Morgan et al., 2012), incident dementia (Holtzer et al., 2008), and probable AD (Brewster et al., 2012). Likewise, it was found to be increased in MCI and AD (Tractenberg and Pietrzak, 2011; MacDonald et al., 2012). Although IIV has been proposed to index cognitive control processes supported by the frontal cortex (MacDonald et al., 2009), accuracy-based IIV across tests representing cognitive control functions (within-domain IIV) to our knowledge has not been examined in healthy control subjects (HCS), MCI, and AD in a single comparative study. Consequently, the aim of our study was to investigate within- and across-domain IIV as markers for prodromal AD. We compared IIV between groups and hypothesized increased levels in MCI and AD. Additionally, and since the apolipoprotein E (APOE) ε4 allele represents a risk factor for late-onset AD (Corder et al., 1993; Petersen et al., 1995; Tang et al., 1996), we explored the relationship between IIV and APOE genotype.

## MATERIALS AND METHODS

### STUDY POPULATION

A total of 267 subjects (HCS *n* = 180, MCI *n* = 44, AD *n* = 26) from on-going studies at the Memory Clinic of the Division of Psychiatry Research and Psychogeriatric Medicine, University of Zurich, were considered for cross-sectional baseline analysis. Participants were recruited from the outpatient population of the Memory Clinic or by advertisement in the local media. All subjects had complete cognitive baseline data acquired between January 2006 and May 2012.

Mild cognitive impairment was diagnosed according to Winblad et al. (2004). AD subjects met NINCDS-ADRDA (McKhann et al., 1984) criteria for probable AD. All diagnoses were made by a multidisciplinary team under the supervision of an experienced psychiatrist. HCS were required to be cognitively healthy and report cognitive well-being. MCI and AD subjects were excluded from the present analyses if there was evidence for the use of psychoactive medication, abuse of alcohol and drugs, other past or present psychiatric or neurological diseases or significant other systemic diseases, or structural abnormalities in the brain assessed by magnetic resonance imaging (MRI) that could account for the cognitive decline. Additionally, HCS were excluded if there was significant medical disease, clinically significant depression or any medication use potentially affecting cognition. Visits included screenings for depression, psychiatric, neurological, and internal diseases, and other disorders that could potentially produce cognitive impairment. The neuropsychological test battery consisted of multiple tests covering the following cognitive domains: episodic memory, executive function, attention/psychomotor processing speed, language, and visual-constructive abilities. Impairment was defined if at least one score per domain was 1.5 SD below group means provided by test-specific normative data.

From the original HCS sample, 31 subjects were excluded from the analyses due to clinically significant neurological disease (*n* = 3), alcohol abuse (*n* = 1), medication (*n* = 25), and due to dropout after the assessment (*n* = 2). Due to a change in test batteries, 13 MCI subjects were missing a test score relevant for computing IIV and were excluded from the analyses. Thus, a total of 149 HCS, 31 MCI patients, and 26 patients with probable AD were eligible for the current analysis of IIV. The final demographic details are presented in **Table 1**. This study was approved by the cantonal ethics committee of canton Zurich, Switzerland, in accordance with the Helsinki Declaration. All participants and/or their legal representatives provided written informed consent prior to study inclusion.

**Table 1:** Demographic information and cognitive measures per diagnostic group.

Characteristic		HCS	MCI	AD	*p* Value
N		149	31	26	N/A
Age, y		68.93 (5.75)	73.39 (5.93)	77.65 (6.10)	0.000*^a,b,c^
Gender, M/F^d^		59/90	11/20	7/19	0.455
Years of education		14.51 (3.25)	12.94 (2.95)	11.12 (2.81)	0.000* ^a,b^
MMSE^e^		29.43 (0.82)	27.87 (1.77)	21.46 (3.78)	0.000* ^a,b,c^
Digit Span Forward	raw	7.49 (1.78)	5.90 (1.33)	5.35 (1.81)	N/A
	res	0.00 (0.99)	-0.63 (0.71)	-0.65 (1.16)	0.000* ^a,b^
Word List Learning	raw	23.70 (2.87)	17.13 (4.49)	12.27 (4.01)	N/A
	res^e^	0.00 (0.99)	-2.25 (1.58)	-3.91 (1.43)	0.000* ^a,b,c^
Category Fluency	raw	23.91 (4.74)	17.71 (5.10)	11.08 (5.25)	N/A
	res	0.00 (0.99)	-1.22 (1.08)	-2.53 (1.15)	0.000* ^a,b,c^
Letter Fluency	raw	31.65 (8.93)	22.58 (10.21)	13.23 (5.96)	N/A
	res	0.00 (0.99)	0.29 (1.52)	-0.66 (1.12)	0.004* ^a,c^
Stroop Trial 3	raw	28.36 (8.70)	35.29 (9.94)	69.04 (48.33)	N/A
	res^e^	0.00 (0.99)	-0.74 (1.01)	-2.74 (2.20)	0.000* ^a,b,c^
Five-Point Test	raw	25.96 (7.00)	18.74 (6.70)	12.19 (5.27)	N/A
	res	0.00 (0.99)	-0.91 (0.87)	-1.70 (0.78)	0.000* ^a,b,c^
Across-domain IIM		0.00 (0.63)	-1.37 (0.82)	-2.36 (0.92)	0.000* ^a,b,c^
Within-domain IIM		0.00 (0.73)	-0.45 (0.86)	-1.70 (0.99)	0.000* ^a,b,c^
Across-domain IIV^f^		0.91 (0.62)	0.98 (0.58)	1.52 (0.75)	0.001* ^a,c^
Within-domain IIV^f^		0.75 (0.55)	1.01 (0.52)	1.23 (0.65)	0.002* ^a,b^

HCS = healthy control subjects; MCI = mild cognitive impairment; AD = Alzheimer’s disease; N/A = not applicable; MMSE = Mini-Mental State Examination; res = standardized residuals; raw = raw scores; IIM = intraindividual mean; IIV = intraindividual variability. Data are means and standard deviations unless specified otherwise. Standardized residuals of cognitive test scores for MCI and AD were calculated by using parameter estimates for age, education and gender in HCS. *Significant global *p* value test by analysis of variance unless specified otherwise. *Post hoc* tests indicated significant differences at *p* ≤ 0.05 or lower (parametric tests) and *p* < 0.01 or lower (non-parametric tests) for ^a^HCS vs. AD; ^b^HCS vs. MCI; ^c^MCI vs. AD. ^d^Pearson’s *_X_*^2^ test. ^e^Kruskal–Wallis test followed by Mann–Whitney test. ^f^Analyses of covariance, means adjusted for age, gender, education and across- or within-domain IIM.

### COMPUTATION OF INTRAINDIVIDUAL VARIABILITY

Considering the clinical applicability of the IIV scores, we retrospectively identified tasks with less than 1% missing values per diagnostic group. Additionally, we only selected tasks with no ceiling or floor effects to prevent suppressing variation at the extreme ends of the distribution.

For calculating across-domain IIV, we used accuracy scores from three tests, each representing a different cognitive domain: Digit Span Forward from the Wechsler Memory Scale-Revised (Härting et al., 2000), assessing verbal short-term memory capacity (Lezak et al., 2004), Word List Learning and Category Fluency from the CERAD-plus test battery (Thalmann et al., 1997) assessing verbal learning and executive function/semantic knowledge, respectively. For calculating within-domain IIV, we used accuracy scores from three tests, each representing executive functions and eliciting recruitment of cognitive control processes. The Letter Fluency test requires participants to name as many words as possible within 3 min while taking into account particular restrictions (i.e., no names, geographically related words, labels, repetitions). Participants need to generate, maintain, and monitor a plan, to select and establish specific responses and, therefore, access cognitive control (Stein et al., 2010; Reiman et al., 2014). Compared with the Category Fluency test, which consists of a single restriction (name animals) and is of a shorter duration (1 min), the Letter Fluency test represents a more complex task. Increasing task complexity is thought to place higher demands on higher order cognitive abilities (Halford et al., 2005) such as cognitive control processes. Accordingly, the Letter Fluency test is thought to rely more on cognitive control processes but less on semantic knowledge than the Category Fluency task (Delis and Kaplan, 2001). Trial 3 from the Stroop Test (Troyer et al., 2006) requires subjects to accurately name the color in which 24 non-congruent color words are printed (i.e., the word blue is printed in red color). Accordingly, participants need to maintain a goal while inhibiting a routine response in favor of a less familiar one, a process which typically involves cognitive control (West et al., 2002). The Five-Point Test (Regard et al., 1982) represents figural fluency and requires participants to draw as many different figures as possible within 3 min by connecting dots displaying the five-dot arrangement on dice. Participants, therefore, need to follow a mental strategy and monitor their performance. This coordination of information to select appropriate behavioral responses represents aspects of cognitive control (Kelemen and Fenton, 2010).

The simplest method to compute IIV is to calculate the intraindividual standard deviation (ISD; Nesselroade and Salthouse, 2004) across each individual’s accuracy scores. Before computing ISD, two missing Stroop Test raw scores in HCS and MCI were imputed with the expected-maximization algorithm in SPSS. Effects associated with age, education, and gender, and potential interactions were estimated from the HCS’ raw scores by using General Linear Model. Parameters for age, education, and gender from this model were used to predict accuracy scores in both MCI and AD subjects. Standardized residuals for MCI and AD were then calculated by subtracting the predicted from the observed accuracy scores and dividing it by the model’s standard error. Residuals from the Stroop Test were log-transformed to achieve normal distribution, and multiplied by -1 to adjust for scaling difference. In sum, this procedure generated standardized residuals representing adjusted accuracy scores with a mean of 0 and variance of ∼1 in HCS. By restricting the variance to ∼1 in HCS, we lowered the risk of overestimating IIV in HCS due to higher mean performance, since ISD is not independent from the mean (Allaire and Marsiske, 2005). Accordingly, residuals deviating from 0 represented adjusted accuracy scores for MCI and AD subjects. We then computed ISD across each individual’s residuals on Digit Span Forward, Word List Learning, and Category Fluency representing across-domain IIV, whereas ISD across residuals on Letter Fluency, Stroop Test, and Five-Point Test represented within-domain IIV. To further address the association between ISD and mean performance, we used the intraindividual mean (IIM) across residuals underlying across-domain IIV (across-domain IIM) and across residuals underlying within-domain IIV (within-domain IIM) as covariates in all relevant analyses.

### GENOTYPING

Apolipoprotein E genotyping was performed by restriction isotyping as described previously (Hixson and Vernier, 1990). For analysis, participants were classified as either carriers (APOE ε2/ε4, ε3/ε4 and ε4/ε4) or non-carriers of the APOE ε4 allele.

### STATISTICS

Group comparisons of normally distributed demographic data, raw and adjusted cognitive data were applied using univariate analysis of variance (ANOVA) followed by Sidak *post hoc* tests correcting for multiple comparisons. Kruskal–Wallis tests followed by Mann-Whitney tests corrected for multiple comparisons were performed to compare not normally distributed variables. Pearson’s chi-square test was used for categorical variables. Univariate analyses of covariance (ANCOVA) with diagnostic group treated as the main effect were performed to evaluate group wise differences in across- and within-domain IIV. Although influences of age, gender and education had already been taken into account while computing IIV, they were used as covariates to control for influences on IIV. Across- and within-domain IIM represented additional covariates. Sum of Square Type III was applied to take into account the unbalanced design. Significant group effects were further examined using Sidak *post hoc* test correcting for multiple comparisons. For parametric analyses, tests were performed with a significance level of *p* < 0.05. Manually correcting for multiple comparisons, a significance level of *p* < 0.017 (0.05/3 = 0.017) was applied for non-parametric analyses. All analyses were performed as two-sided tests by using the statistical analysis software package PASW 19.0 for Windows.

## RESULTS

Descriptive statistics for demographic information and adjusted cognitive data, as well as cognitive raw data is listed in **Table 1**.

**FIGURE 1:**
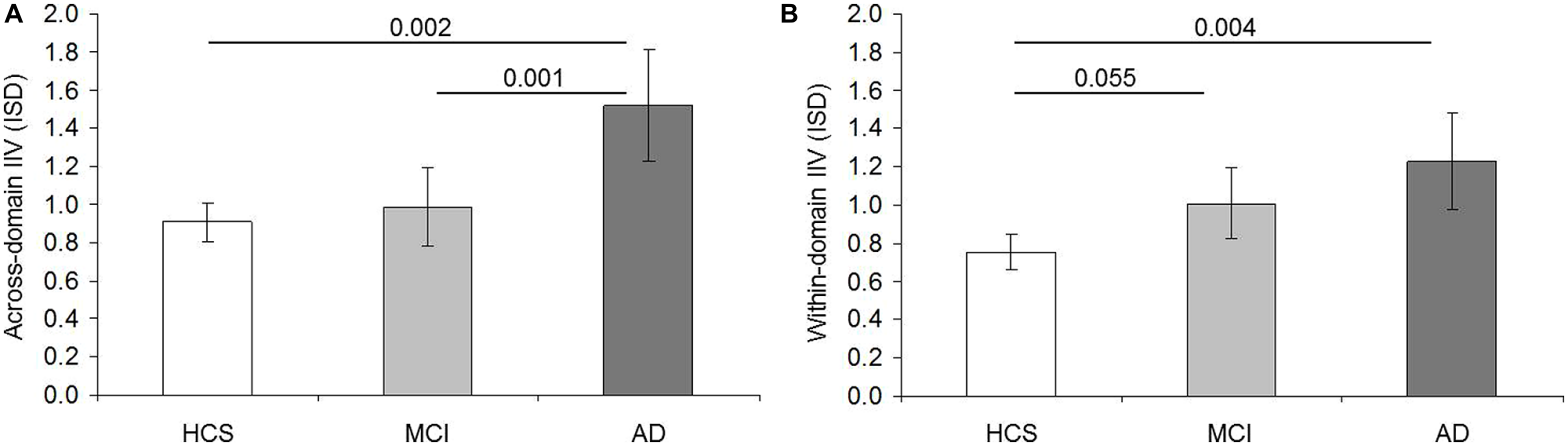
**Comparison of intraindividual variability (IIV) scores between diagnostic groups.** Intraindividual standard deviation (ISD) representing mean across-domain IIV **(A)** and mean within-domain IIV **(B)** per diagnostic group (HCS = healthy control subjects; MCI = mild cognitive impairment; AD = Alzheimer’s disease). Error bars display 95% confidence interval for the mean. Pairwise *p* values shown are based on Sidak *post hoc* tests following analyses of covariance for the comparison of means adjusted for age, years of education, gender, as well as mean across-domain performance **(A)** and mean within-domain performance **(B)**, respectively.

Across-domain IIV was influenced by age [*F*(1,199) = 3.958; *p* = 0.048; $\eta_{p}^{2}$ = 0.020], and slightly by across-domain IIM [*F*(1,199) = 3.520; *p* = 0.062; $\eta_{p}^{2}$ = 0.017] but not by education [*F*(1,199) = 0.076; *p* = 0.783; $\eta_{p}^{2}$ = 0.000] or gender [*F*(1,199) = 1.346; *p* = 0.247; $\eta_{p}^{2}$ = 0.007]. But first and foremost we observed a main effect between the diagnostic groups [*F*(2,199) = 7.310; *p* = 0.001; $\eta_{p}^{2}$ = 0.068]. Patients with AD revealed higher IIV than HCS (*p* = 0.002; 95% CI = 0.192–1.030) and MCI (*p* = 0.001; 95% CI = 0.170–0.892), whereas IIV did not differ between MCI and HCS (*p* = 0.896; 95% CI = –0.226–0.387; **Figure 1A**). Within-domain IIV was not influenced by age [*F*(1,199) = 0.054; *p* = 0.816; $\eta_{p}^{2}$ = 0.000], education [*F*(1,199) = 2.237; *p* = 0.136; $\eta_{p}^{2}$ = 0.011), gender [*F*(1,199) = 2.613; *p* = 0.108; $\eta_{p}^{2}$ = 0.013] or within-domain IIM [*F*(1,199) = 1.500; *p* = 0.222; $\eta_{p}^{2}$ = 0.007], but differed among diagnostic groups [*F*(2,199) = 6.330; *p* = 0.002; $\eta_{p}^{2}$ = 0.060]. IIV was higher in AD than HCS (*p* = 0.004; 95% CI = 0.126–0.825). But contrary to across-domain IIV, within-domain IIV was similar in AD and MCI (*p* = 0.374; 95% CI = 0.142–0.582). More importantly, there was a strong trend for higher IIV in MCI than in HCS (*p* = 0.055; 95% CI = –0.004–0.514; **Figure 1B**).

To evaluate whether there was a relationship between IIV scores, a difference score was calculated by subtracting within- from across-domain IIV. ANCOVA was performed by treating age, education, gender, and across- and within-domain IIM as covariates. The effect of diagnostic group did not approach significance [*F*(2,198) = 1.497; *p* = 0.226; $\eta_{p}^{2}$ = 0.015], indicating similar differences between IIV scores across diagnostic groups. The qualitative analysis of difference scores, however, revealed positive difference scores within each group (HCS: *M* = 0.141, SD = 0.805; MCI: *M* = 0.005, SD = 0.757; AD: *M* = 0.340, SD = 0.989), indicating a tendency toward higher across- than within-domain IIV in these groups.

The relationship between APOE status and IIV was explored in a subsample with available genotype. Descriptive statistics for demographic information and adjusted cognitive data is listed in **Table 2**.

**Table 2:** Demographic information and cognitive measures per diagnostic group and APOE genotype.

Characteristic	HCS		MCI		AD		*p* Value
APOE status	ε4+	ε4-	ε4+	ε4-	ε4+	ε4-	N/A
N^c^	26	87	16	14	9	14	0.004*
Age, y	71.04 (5.92)	68.89 (5.72)	73.31 (6.44)	73.36 (5.75)	77.33 (3.87)	78.07 (7.43)	-
Gender, M/F^d^	11/15	39/48	2/14	9/5	4/5	3/11	0.003*^a^
Years of education	14.77 (2.89)	14.72 (3.52)	12.44 (2.78)	13.57 (3.23)	10.67 (2.18)	10.93 (3.17)	-
MMSE	29.23 (0.91)	29.47 (0.83)	27.44 (1.50)	28.21 (1.97)	20.56 (3.40)	21.86 (4.11)	-
Digit Span forward	0.02 (1.14)	-0.008 (1.03)	-0.61 (0.67)	-0.58 (0.74)	-0.37 (1.34)	-0.57 (0.99)	-
Word List Learning	-0.06 (0.94)	0.05 (1.07)	-2.23 (1.61)	-2.14 (1.58)	-3.78 (1.68)	-3.99 (1.47)	-
Category Fluency	0.33 (0.99)	-0.06 (1.01)	-1.37 (0.94)	-0.89 (1.11)	-2.39 (1.42)	-2.63 (1.04)	-
Letter Fluency	0.57 (1.16)	-0.11 (0.88)	0.65 (1.13)	-0.17 (1.83)	-1.29 (1.33)	-0.39 (0.88)	0.002*^b^
Stroop Trial 3	0.02 (0.94)	0.07 (1.04)	-0.94 (0.86)	-0.44 (1.12)	-2.78 (2.01)	-2.95 (2.45)	-
Five-Point Test	0.25 (1.00)	-0.05 (0.97)	-1.03 (0.61)	-0.77 (1.12)	-1.71 (0.97)	-1.77 (0.75)	-
Across-domain IIM	0.09 (0.61)	0.00 (0.68)	-1.41 (0.80)	-1.20 (0.77)	-2.18 (1.26)	-2.39 (0.77)	-
Within-domain IIM	0.28 (0.72)	-0.02 (0.72)	-0.44 (0.64)	-0.46 (1.12)	-1.93 (1.01)	-1.70 (1.00)	-
Across-domain IIV^e^	0.91 (0.46)	0.84 (0.44)	1.03 (0.81)	1.18 (0.22)	1.86 (0.59)	1.84 (0.87)	-
Within-domain IIV^e^	0.86 (0.40)	0.68 (0.41)	0.97 (0.47)	1.08 (0.05)	1.33 (0.72)	1.51 (1.22)	0.049*^b^

HCS = healthy control subjects; MCI = mild cognitive impairment; AD = Alzheimer’s disease; ε4+ = ε4 carrier; ε4- = ε4 non-carrier; N/A = not applicable; MMSE = Mini-Mental State Examination; IIM = intraindividual mean; IIV = intraindividual variability. Data are means and standard deviations unless specified otherwise. Standardized residuals of cognitive test scores for MCI and AD were calculated by using parameter estimates for age, education and gender in HCS. * If not stated otherwise: Significant *p* value test by analysis of variance per diagnostic group indicated significant differences at *p* < 0.05 or lower between ε4 carrier and ε4 non-carrier in ^a^MCI, ^b^HCS. – indicate no significant group differences. ^c^Pearson’s *_X_*^2^ test across diagnostic groups. ^d^Pearson’s *_X_*^2^ test per diagnostic group. ^e^Analysis of covariance in MCI, means adjusted for gender.

To compare IIV scores between ε4 carrier and non-carrier within each group, we performed ANCOVAs by treating gender as a covariate in MCI. Across-domain IIV did not vary with APOE status in HCS [*F*(1,111) = 0.368; *p* = 0.545; $\eta_{p}^{2}$ = 0.003], MCI [*F*(1,27) = 0.227; *p* = 0.638; $\eta_{p}^{2}$ = 0.008] or AD [*F*(1,21) = 0.003; *p* = 0.957; $\eta_{p}^{2}$ = 0.000]. Likewise, within-domain IIV did not vary as a function of APOE status in MCI [*F*(1,27) = 0.348; *p* = 0.560; $\eta_{p}^{2}$ = 0.013] or AD [*F*(1,21) = 0.149; *p* = 0.703; $\eta_{p}^{2}$ = 0.007]. In HCS, however, there was a weak though significant effect of APOE status [*F*(1,111) = 3.972; *p* = 0.049; $\eta_{p}^{2}$ = 0.035] indicating increased within-domain IIV in ε4 carrier.

## DISCUSSION

This study examined whether two different accuracy-based IIV measures on established neuropsychological tasks differed between HCS, MCI, and AD. Our results suggest an increasing breakdown of cognitive control functions early in prodromal AD resulting in increased IIV. More precisely, across- and within-domain IIV, as used in the present study, may differ from each other as a function of cognitive control required by the underlying tasks. Within-domain IIV tapping cognitive control functions more closely was increased in AD and MCI vs. HCS, and appears to constitute a potential marker for the detection of prodromal AD at the MCI stage. Across-domain IIV tapping less cognitive control functions was increased in AD vs. MCI and HCS, and may detect incipient dementia and separate AD from the MCI stage.

The establishment of cognitive markers that accurately predict the diagnosis of AD and its preclinical manifestation MCI, supports the effort of early detection. Mean performance in tests of verbal episodic memory (Derby et al., 2013) and executive function (Schroeter et al., 2012) in particular are known markers for predicting AD. The reliable detection of early cognitive impairment based on mean cognitive performance in clinical routine, however, poses a challenge. Most importantly, cognitive changes in subjects with high educational background may be present prior to a clinical diagnosis but may be very subtle, and therefore may be undetected. Cognitive measures that discriminate between MCI due to AD and HCS based on abilities relevant to everyday life (i.e., Bird et al., 2010) might further support the reliable early detection, but such tests have not been under ample investigation. Across- and within-domain IIV was found to be independent from the mean cognitive performance, and might therefore represent a more sensitive early marker of cognitive impairment than mean cognitive performance.

Although it represents an easy to use measure in clinical routine (Holtzer et al., 2008) only few studies have investigated IIV across accuracy scores from tests representing different cognitive domains in HCS and AD (Lindenberger and Baltes, 1997; Christensen et al., 1999; Brewster et al., 2012; MacDonald et al., 2012) or in HCS, MCI and AD (Tractenberg and Pietrzak, 2011). Even though IIV has been related to impaired cognitive control functions (MacDonald et al., 2009), we are not aware of any study investigating IIV across accuracy scores from tests uniquely representing cognitive control in these groups. Latency-based IIV has been suggested to be a more sensitive measure than accuracy-based IIV (Hultsch et al., 2000), and the direct comparison of our results with work on latency-based IIV is challenging. However, different studies have demonstrated a relationship between these measures (Hultsch et al., 2002; Hilborn et al., 2009). Accordingly, increased latency- and accuracy-based IIV have been linked to older age (Hultsch et al., 2002; West et al., 2002; Lövdén et al., 2007; Hilborn et al., 2009), cognitive decline (Kliegel and Sliwinski, 2004; Lövdén et al., 2007), and to predict probable AD (Brewster et al., 2012) and incident dementia (Holtzer et al., 2008). IIV has therefore widely been accepted as a stable trait (MacDonald et al., 2006) - possibly reflecting central nervous system integrity (MacDonald et al., 2009). More precisely, evidence for a strong association between IIV and frontal gray and white matter integrity (Stuss et al., 2003; Lövdén et al., 2013), and evidence of changed gray and white matter integrity in MCI and AD (Jackson et al., 2012; Yang et al., 2012; Radanovic et al., 2013) support the idea of frontal system disruptions underlying increased IIV in dementia (Jackson et al., 2012).

It is beyond the aim of our study to draw direct inferences about the origins of IIV. However, consistent with our hypothesis and the literature (Tractenberg and Pietrzak, 2011; MacDonald et al., 2012), we found increased across-domain IIV in AD vs. HCS and in AD vs. MCI. Therefore, across-domain IIV was similar in MCI and HCS. Even though others have examined latency-based IIV within but not accuracy-based IIV across non-cognitive control tasks (Tales et al., 2012), they have also reported similar IIV in these groups. Moreover, MCI subjects who later converted to dementia were found to have higher IIV than non-converters. The absence of a group difference between MCI and HCS in our study may therefore be related to a low proportion of future converters in our MCI group. Additionally, and since higher IIV has been found in tasks requiring cognitive control (MacDonald et al., 2009), the requirement of cognitive control processes in the tasks underlying across-domain IIV might have been too limited to differentiate between these groups. Consistent with this assumption, and consistent with the literature on latency-based within-domain IIV (Duchek et al., 2009) we found within-domain IIV, and hence IIV across tasks placing more demands on cognitive control processes, being increased in MCI vs. HCS. Considering impaired cognitive control functions in MCI and AD (Schroeter et al., 2012), one might have expected increased within-domain IIV in AD vs. MCI. Against our expectations it was similar in both groups. Since the use of a high number of trials has previously been proposed to reliably detect IIV (Schmiedek et al., 2009), it might be less pronounced when computed across three accuracy scores only, even when computed across cognitively demanding tasks as in our study.

The similar difference scores in all groups offer additional support for increasing accuracy-based IIV across groups in general. Though not significantly different from the difference scores in HCS and AD, it was very small in MCI (*M* = 0.005), reflecting the increase in within-domain IIV/stability in across-domain IIV between HCS and MCI. Due to the dependence of IIV on cognitive control tasks, higher within- than across-domain IIV may be expected. However, the higher across-domain IIV might be caused by the early deterioration of episodic memory (Albert et al., 2011) and short-term memory capacity (Schmitt et al., 2009) compared to other cognitive domains in the disease process. Consequently, and although mean performance was considered in the relevant analyses, the use of a verbal learning task and a task assessing short-term memory capacity might have triggered higher across-domain IIV.

Additionally, we found increased within-domain IIV in HCS ε4 carrier vs. non-carrier, whereas there was no ε4-related change in IIV in the other groups. Our result is consistent with findings from Duchek et al. (2009) who have reported increased latency-based IIV in a cognitive control task, but similar IIV in tasks without cognitive control components in HCS ε4 carrier vs. non-carrier. Since the frontal lobe constitutes a brain region that manifests ε4-effects even early in the disease (Filbey et al., 2010), and is thought to be at the basis of IIV (MacDonald et al., 2009), the present findings offer further support for a relationship between within-domain IIV and APOE status. It may, however, well be that ε4-related change in IIV appears in HCS but may not be evident by the MCI and AD stage.

The major limitation of our study is related to the selection of the tasks. The limited number of available neuropsychological tests did not allow applying factor analyses. Hence, the tasks and their domain-relatedness were identified following the literature. Based on the high engagement of cognitive control processes, we identified executive function tasks to calculate within-domain IIV. However, cognitive control processes affect a wide range of cognitive functions. This is why we aimed to identify tasks placing low demands on cognitive control for across-domain IIV, and tasks placing high demands on cognitive control for within-domain IIV. This approach, however, revealed potential confounding factors which make it difficult to clearly determine whether our results can be attributed to the fact that IIV was calculated across vs. within-domain, or to the fact that the underlying tasks elicited low vs. high cognitive control. However, a higher number as well as a wider range of neuropsychological tests would be required to clearly differentiate between these aspects. Hence, only cautious conclusions can be drawn based on our results. Both aspects might be considered relevant with regard to across-domain IIV. More precisely, similar across-domain IIV between HCS and MCI is most likely based on equally reduced cognitive abilities across domains in MCI (see **Table 1**). Although cognitive control processes are expected to be impaired in prodromal AD, the low level of cognitive control processes elicited by these tasks may have been responsible for the uniformity of the decrease. The impairment of cognitive control processes, however, may have been sufficient to produce variation across tasks in AD (e.g., unequally decreased test performances in AD vs. MCI, see **Table 1**). In contrast, the aspect of high vs. low cognitive control might be considered relevant with regard to within-domain IIV. Increased within-domain IIV in MCI is most likely based on unequally decreased performances in within-domain IIV tasks (see **Table 1**). If it was the across vs. within-domain aspect that was critical, equally decreased performances could have been expected. Impaired cognitive control processes producing inconsistencies across performances in cognitive control sensitive tasks, and hence, producing higher within-domain IIV in MCI seem more plausible. The further reduction of cognitive control abilities in AD might lead to two different scenarios: a) further increased within-domain IIV due to inconsistent test performances or b) reduced within-domain IIV based on floor effects. Since tasks with potential floor effects were excluded, the latter does rather not apply. Although within-domain IIV did not differ significantly between MCI and AD, IIV was higher in AD (**Figure 1**), indicating further increasing IIV. The lack of a significant difference might indeed have been caused by the low sample size, and by the very subtle characteristic of within-domain IIV in general. The finding of higher across- than within-domain IIV across the groups in turn is most likely related to inconsistent performances across tests representing different cognitive domains. In summary, and although the present results must be interpreted with caution, our results indicate that the aspect of across vs. within domain might be most relevant for the general characteristic of the IIV scores (higher across- than within-domain IIV). In contrast, the aspect of high vs. low cognitive control might be at the basis of within-domain IIV group differences.

The minor limitations of our study are attributed to the cross-sectional design. Our results, therefore, do not permit to claim causality regarding the relationship between AD pathology and IIV. More precisely, it has been argued that cross-sectional data do not permit to clearly distinguish variability caused by aging or neurodegeneration from stable individual characteristics (Lindenberger and Potter, 1998). This risk was addressed by treating age and within- and across-domain IIM as covariates in all analyses. Furthermore, most test performances underlying IIV were also used for diagnostic purpose, thus posing the risk of circularity. However, we assume the risk to be minimal, since the outcome of interest in the present study was the ISD calculated across tasks. In addition to that, neuropsychological tasks that had not been used for IIV calculation, wide-ranging medical information, and clinical evaluation also contributed to the diagnosis. Another limitation is related to the multidimensional nature of the neuropsychological tasks. Although the tasks which were used to calculate within-domain IIV place high demands on cognitive control processes, they do not exclusively assess this particular cognitive function. Processing speed (Greenaway et al., 2009), inhibition (Troyer et al., 2006) and visuo-construction (Lezak et al., 2004) represent further cognitive abilities that are crucial for successfully performing the Letter Fluency task, the Stroop Test, and the Five-Point Test, respectively. They might, therefore, represent potential confounders in the present study. Since the tasks which were used to calculate across-domain IIV, however, place fewer demands on cognitive control processes than the task which were used to calculate within-domain IIV, we assume this risk to be reduced.

Despite these limitations, and although comparison with other studies may be limited due to methodological differences among studies (e.g., IIV definition and measures, diagnostic criteria), the present study offers further support for increased IIV in MCI and AD in general, and for increased accuracy-based IIV in particular. From a clinical point of view, accuracy-based IIV may be more useful than latency-based IIV measures in everyday clinical routine. First, tasks assessing cognitive control functions and non-cognitive control functions are usually included in standard clinical neuropsychological test batteries, and therefore allow IIV calculations without applying additional tests. Second, assessing accuracy-based IIV avoids the necessity to add multiple trials or blocks of the same task to the standard test battery, and therefore reduces the burden for the patients in dementia diagnostics. The present study, therefore, underscores the importance of considering the value of IIV in the early detection of prodromal AD and demonstrates the usability of accuracy-based IIV measures in AD diagnosis. Both, across- and within-domain IIV may represent potential cognitive markers for the early detection of prodromal AD. However, further examination by using a higher number of more complex tests in a longitudinal design is needed to provide more specific information about the predictive value of these IIV scores.

## AUTHOR CONTRIBUTIONS

Research questions and study design were formulated by Andrea M. Kälin, Anton F. Gietl, Christoph Hock. Data acquisition was carried out by Andrea M. Kälin, Anton F. Gietl, Florian Riese. Statistical analyses were performed by Andrea M. Kälin, Marlon Pflüger, Lutz Jäncke. All authors contributed to the interpretation of data and to the manuscript drafting, writing, and revising.

## Conflict of Interest Statement

The authors declare that the research was conducted in the absence of any commercial or financial relationships that could be construed as a potential conflict of interest.
